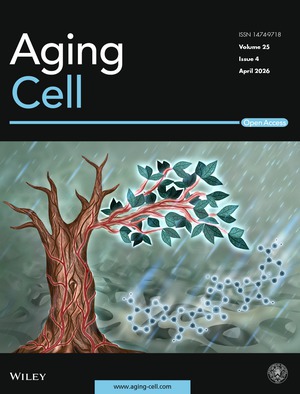# Additional Cover

**DOI:** 10.1111/acel.70491

**Published:** 2026-04-18

**Authors:** Jianfeng Yu, Mingzhuang Hou, Yaoge Deng, Chenqi Yu, Yang Liu, Kang Kang, Xiaowei Xia, Xiaoping Li, Huilin Yang, Dinghua Jiang, Wu Xu, Yijian Zhang, Xuesong Zhu

## Abstract

The cover image is based on the article *Double‐Pronged NAD Preservation: Delaying Cellular Senescence and Initiating Musculoskeletal Regeneration* by Jianfeng Yu et al., https://doi.org/10.1111/acel.70468.